# A successful shift from thoracotomy to video-assisted thoracoscopic lobectomy for non-small cell lung cancer in a low-volume center

**DOI:** 10.1093/icvts/ivae018

**Published:** 2024-01-30

**Authors:** Viktor Asbjornsson, Gyda Johannsdottir, Daniel Myer, Thorri Geir Runarsson, Leon Arnar Heitmann, Gudrun N Oskarsdottir, Per Martin Silverborn, Henrik Jessen Hansen, Tomas Gudbjartsson

**Affiliations:** Faculty of Medicine, University of Iceland, Reykjavik, Iceland; Department of Cardiothoracic Surgery, Landspitali University Hospital, Reykjavik, Iceland; Faculty of Medicine, University of Iceland, Reykjavik, Iceland; Faculty of Medicine, University of Iceland, Reykjavik, Iceland; Faculty of Medicine, University of Iceland, Reykjavik, Iceland; Faculty of Medicine, University of Iceland, Reykjavik, Iceland; Department of Pulmonology, Landspitali University Hospital, Reykjavik, Iceland; Department of Cardiothoracic Surgery, Landspitali University Hospital, Reykjavik, Iceland; Department of Cardiothoracic Surgery, Sahlgrenska University Hospital, Gothenburg, Sweden; Department of Cardiothoracic Surgery, Rigshospitalet University Hospital, Copenhagen, Denmark; Faculty of Medicine, University of Iceland, Reykjavik, Iceland; Department of Cardiothoracic Surgery, Landspitali University Hospital, Reykjavik, Iceland

**Keywords:** Video-assisted thoracoscopic surgery, Lobectomy, Non-small-cell lung cancer, Learning curve, 30-Day mortality, Conversion

## Abstract

**OBJECTIVES:**

Although video-assisted thoracoscopic surgery (VATS) lobectomy has become the gold standard for pulmonary resections of non-small-cell lung cancer (NSCLC), lobectomy is still performed via thoracotomy in many European and North American centres. VATS lobectomy was implemented overnight from thoracotomy in our low-volume centre in early 2019, after 1 senior surgeon undertook observership VATS-training overseas, and immediately became the mainstay of surgical treatment for NSCLC in Iceland. We aimed to investigate our short-term outcomes of VATS lobectomy.

**METHODS:**

This was a retrospective study on all pulmonary resections for NSCLC in Iceland 2019–2022, especially focusing on VATS lobectomies, all at cTNM stage I or II. Data were retrieved from hospital charts, including information on perioperative complications, mortality, length of stay and operation time.

**RESULTS:**

Out of 204 pulmonary resections, mostly performed by a single senior cardiothoracic surgeon, 169 were lobectomies (82.9%) with 147 out of 169 (87.0%) being VATS lobectomies. Anterolateral thoracotomy was used in 34 cases (16.7%), including 22 lobectomies (64.7%), and 5 (3.4%) conversions from VATS lobectomy. The median postoperative stay for VATS lobectomy was 4 days and the average operating time decreased from 155 to 124 min between the first and last year of the study (*P* < 0.001). The rate of major and minor complications was 2.7% and 15.6% respectively. One year survival was 95.6% and all patients survived 30 days postoperatively.

**CONCLUSIONS:**

The implementation of VATS lobectomy has been successful in our small geographically isolated centre, serving a population of 390 000. Although technically challenging, VATS lobectomy was implemented fast for most NSCLC cases, with short-term outcomes that are comparable to larger high-volume centres.

**Figure:**
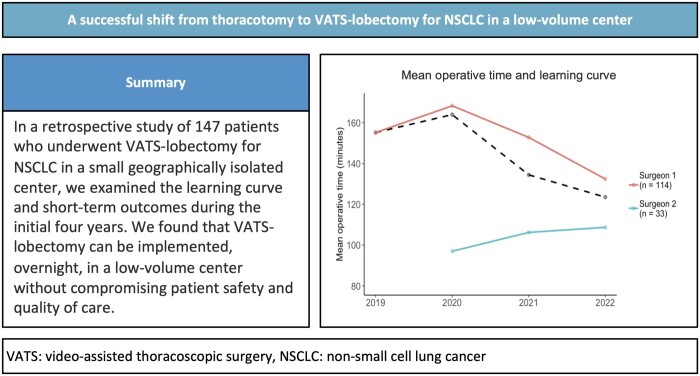

## INTRODUCTION

The mainstay of curative treatment for non-small-cell lung cancer (NSCLC) is lobectomy and is used for around 80% of the surgically treated patients [1, 2]. Traditionally lobectomies were performed via thoracotomy. However, for the last 3 decades, video-assisted thoracoscopic surgery (VATS) lobectomy has gained increasing popularity for showing advantages over thoracotomy regarding hospital stay, fewer complications, faster recovery times and non-inferior survival rates both for early-stage and locally advanced disease [3–6]. Despite these clinical benefits ∼30–40% of centres in North America and Europe still perform lobectomy via thoracotomy [7, 8]. Globally the implementation of VATS is challenged both by being a more expensive option and more technically challenging, reflected in a learning curve ranging of 35–50 procedures [6, 8–11].

In Iceland, a geographically isolated island in the North Atlantic with around 390 000 inhabitants, lung cancer has for decades been the second most common cancer for both genders [12]. Today, ∼170 individuals are diagnosed annually with NSCLC, with every third patient undergoing pulmonary resection at Landspitali University Hospital, the only centre offering pulmonary surgery in Iceland [13]. Traditionally, these procedures were performed by muscle-sparing anterolateral thoracotomy by senior cardiothoracic surgeons not having previous experience with VATS lobectomy, with a 30-day mortality under 1% [2, 14]. In early 2019, VATS was introduced as the mainstay treatment for both lobectomies and sublobar resections in our centre, after one of the senior surgeons (surgeon 1) undertook a 2-month VATS-training programme, mostly consisting of observership, at high-volume centres in Sweden and Denmark. His time overseas was mostly spent observing, however, also performing several VATS lobectomies under the supervision of another senior surgeon (surgeon 2) that a year later moved to Iceland to work partime.

The aim of this study was to investigate short-term outcomes of our initial 4-year experience with VATS lobectomies, focusing on the learning curve, postoperative complications and 30-day mortality.

## MATERIALS AND METHODS

### Ethics statement

The study was approved by the Icelandic National Bioethics Committee (Ref.: 98-060-V5-S1). As individual patients were not identified, the need for individual consent was waived.

### Study design

This was a retrospective cohort study including all patients who underwent lobectomy with VATS for primary NSCLC with curative intent in Iceland from 1 January 2019 until 31 December 2022. The inclusion criteria for VATS lobectomy were histologically verified NSCLC, clinical TNM stages I or II, all tumours being <7 cm in maximal diameter and without signs of mediastinal lymph node metastasis (N0) on imaging studies. Endobronchial ultrasound was performed if mediastinal lymph nodes were positive on positron emission tomography or enlarged. Otherwise a lobectomy was performed on resectable patients via an anterolateral thoracotomy. This study focused on the 147 patients operated on with VATS lobectomy, including those who were converted to thoracotomy, but excluding patients who underwent lobectomy via anterolateral thoracotomy incision, wedge resections, segmentectomy and pneumonectomy. Patients with a postoperative pathological diagnosis of carcinoma *in situ*, adenoid cystic carcinoma, mucoepidermoid carcinoma, carcinoid or sarcoma were excluded.

All procedures were performed at Landspitali University Hospital, the only hospital in Iceland performing cardiothoracic surgery. Two senior surgeons conducted all procedures, both being senior cardiothoracic surgeons performing pulmonary and cardiac procedures: one of them (surgeon 1) had no prior experience with VATS lobectomy and performed 78% of the cases, including all cases performed in the first year of the study, but the other (surgeon 2) had 7 years experience with VATS lobectomy when starting working part time in Iceland in 2020.

### Data curation and demographics

Cases were identified from 3 databases: the operation registry at Landspitali University Hospital, the diagnosis registry at Landspitali University Hospital and the Icelandic Cancer Registry.

Clinical data were retrieved from medical records and surgical reports. Over 60 variables were collected for each patient, including age, gender, smoking history, comorbidity (chronic obstructive pulmonary disease, ischaemic heart disease and arrhythmias), pulmonary function tests (forced expiratory volume in 1 s and forced vital capacity), type of surgery performed, postoperative length of stay, postoperative complications, adjuvant therapy, American society of anaesthesiology score and date of death.

The seventh edition of the tumor, node, metastasis (TNM) staging system was used to stage all the tumours postoperatively (pTNM). Previous smokers were defined as patients who stopped smoking 5 years before surgery. Complications following surgery were defined as major and minor complications. Major complications included bronchopleural fistula, myocardial infarction, acute respiratory distress syndrome and reoperation for postoperative bleeding. Minor complications were congestive heart failure, empyema, new-onset atrial fibrillation, postoperative pneumonia, recurrent nerve paralysis, wound infection, air leakage over 7 days and intraoperative bleeding of >1 l (without reoperation). Operation mortality was defined as death within 30 days of surgery. Patients were assigned a date of death or identified as living on 14 June 2023, using data from the Icelandic National Population Registry. The mean follow-up time was 795 days (26.1 months, range: 65–1609 days).

### Surgical procedure

VATS lobectomy was performed in general anaesthesia using double-lumen intubation for single lung ventilation, with the patient placed in the lateral decubitus position. The standard three-port anterior approach was used. A 3- to 5-cm utility incision was performed in the mid-axillary line between the 4th and 5th ribs, without using a rib retractor. A 0.5- or 1-cm camera port was placed at the level of the diaphragm in the anterior axillary line, with an additional 1.5 cm in the posterior axillary line, at the same level as the camera port. The utility incision allowed for direct access to the lung hilum and for dissection of vessels and bronchi. In case of conversion, the utility incision was extended to an anterolateral thoracotomy. Lobectomy was always performed with an anatomical dissection of the hilum and systematic mediastinal lymph node dissection was performed, including stations 4R, often 2R, 7, 10, 11 and 9 on the right side and stations 4L, 5, 10, 11, 7 and 9 on the left side. The pulmonary veins, arteries and bronchi were dissected and stapled using an endoscopic stapler, and an endoscopic bag was used to remove the specimens through the utility incision. Before closing the incision, an intercostal local anaesthesia from the thoracic cavity was performed using ropivacaine.

### Statistical analysis

Microsoft Excel was used to store the dataset and statistical analyses were carried out using R (Vienna, Austria), version 4.2.2., via R Studio (RStudio, PBC, USA), version 2022.12.0 + 353. Chi-squared test was used to compare categorical variables and analysis of variance was used to compare continuous variables. Differences were considered significant if the *P*-value was <0.05. The Kaplan–Meier method was used to estimate the overall survival of the group.

## RESULTS

**Figure 1:**
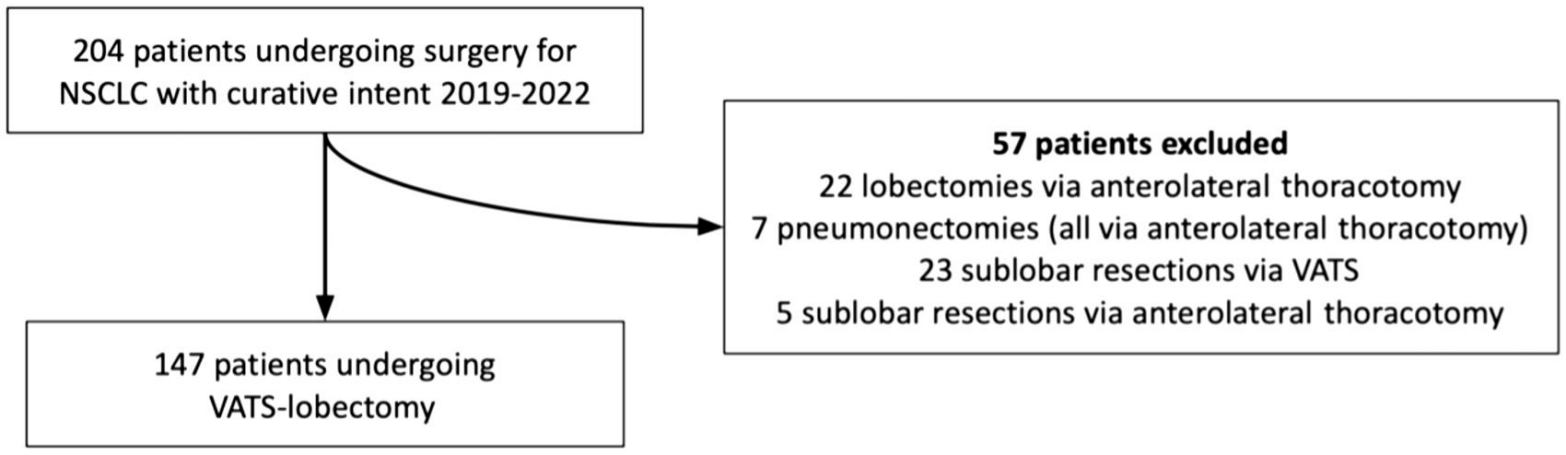
Study inclusion flowchart. NSCLC: non-small-cell lung cancer; VATS: video-assisted thoracoscopic surgery.

A flowchart showing the inclusions of the patients is shown in Fig. 1. Out of 621 patients who were diagnosed with NSCLC during the 4-year study period, 197 (31.7%) of them underwent 204 pulmonary resections. Lobectomy was performed in 169 cases (82.9%), with 147 out of 169 (87.0%) being VATS lobectomies and 22 via anterolateral thoracotomy incision (13.0%). Other pulmonary resections consisted of 28 sublobar resections (wedge or segmentectomy, 13.7%), 23 performed with the VATS technique and 7 pneumonectomies all performed via a thoracotomy incision (3.4%).

Patient demographics, comorbidities and risk factors are shown in Table 1. The average age was 70 [standard deviation (SD) = 8] years and 54.4% were female. Altogether, 92.5% of the patients had a history of smoking with 36 (SD = 24) pack-years on average and 53.7% smoked within 5 years prior to the diagnosis. For TNM staging, the majority exhibited stage I (71.4%) disease, predominantly stage IA (46.9%). Stage II disease was seen in 22.4% of cases, with stage IIA and IIB rates being 8.8% and 13.6% respectively. Additionally, 6.2% of patients had surgically resectable locally advanced disease (stage IIIA). During the study period, 10 patients had N1 lymph node disease after pathology analysis and 5 patients N2 disease. The most common histology types were adenocarcinoma (76.2%) and squamous cell carcinoma (20.4%), but large cell and adenosquamous carcinoma were less common (1.4% and 2.0%, respectively). Cancer-free surgical margins were observed in 146 cases (99.3%) and microscopic disease at the resection margins (positive margins) was detected in 1 patient (0.7%). The mean size of the surgically resected tumours measured 2.8 (SD = 1.6) cm, with a range spanning from 0.9 to 9.8 cm.

**Table 1:** Patient demographics, preoperative risk factors and comorbidities, TNM staging, histology, post-operative complications and adjuvant therapy administered for the first 147 video-assisted thoracoscopic surgery lobectomies in Iceland, 2019–2022

	*n* (%)
Descriptive factors	
Female	80 (54.4)
Age (years)	70 (SD = 8)
Risk factors	
Smoking history	136 (92.5)
Smoker within 5 years	79 (53.7)
COPD	56 (38.1)
IHD	37 (25.2)
Arrhythmias	31 (21.1)
FEV1 < 75%	37 (25.2)
pTNM stage	
IA	69 (46.9)
IB	36 (24.5)
IIA	13 (8.8)
IIB	20 (13.6)
IIIA	9 (6.2)
Tumour factors	
Adenocarcinoma	112 (76.2)
Squamous cell carcinoma	30 (20.4)
Adenosquamous carcinoma	3 (2.0)
Large cell carcinoma	2 (1.4)
Diameter of tumour (mean, cm)	2.8 (range: 0.9-9.8)
Free surgical margins	146 (99.3)
Major complications	4 (2.7)
BPF	0
MI	2 (1.4)
ARDS	1 (0.7)
Reoperation for bleeding	2 (1.4)
Minor complications	23 (15.6)
CHF	1 (0.7)
Empyema	1 (0.7)
AF	6 (4.1)
Pneumonia	2 (1.4)
RNP	0
Wound infection	0
Air leakage >7 days	13 (8.8)
Intraoperative bleeding >1 l	3 (2.0)
Adjuvant therapy	25 (17.0)
Median days until administration	41 [IQR: 35-48]
Total postoperative stay	
Mean	5.9 (SD = 5.3)
Median	4 [IQR: 2-7]

AF: atrial fibrillation; ARDS: acute respiratory distress syndrome; BPF: bronchopleural fistula; CHF: congestive heart failure; COPD: chronic obstructive pulmonary disease; FEV1: forced expiratory volume in 1 s; IHD: ischaemic heart disease; MI: myocardial infarction; RNP: recurrent nerve paralysis; SD: standard deviation.

Both minor and major complications are listed in Table 1. Four patients (2.7%) had 1 or more major complications. No patient developed a bronchopleural fistula. Altogether, 23 patients (15.6%) had 1 or more minor complications, air leakage for >7 days being the most common one (13 patients, 8.8%), with 19 patients (12.9%) having air leakage for >5 days. Atrial fibrillation occurred in 4.1% of patients and other minor complications were less common, which included intraoperative bleeding over 1000 ml (2.0%), pneumonia (1.4%) and empyema (0.7%). The median postoperative stay was 4 days overall. Adjuvant therapy was administered postoperatively to 25 patients (17.0%) and the median days until the start of adjuvant therapy was 41 days.

The annual proportions of lobectomies performed with the VATS technique from 2019 to 2022, were 80.5% (*n* = 33), 82.5% (*n* = 33), 89.2% (*n* = 33) and 94.1% (*n* = 48), respectively, but did not change significantly (*P* = 0.125). In 5 patients (3.4%) VATS was converted to thoracotomy because of intraoperative bleeding (*n* = 2) or adhesions (*n* = 3), all of them occurring in the first 2 years of the study.

**Figure 2:**
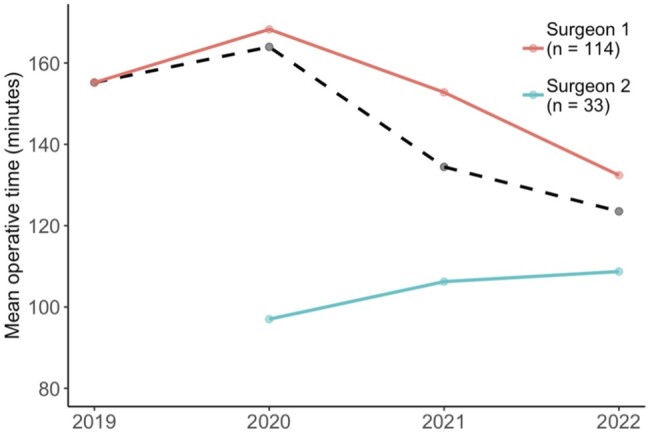
The mean operative time (dashed line) for each year and for the 2 surgeons that performed the first 147 VATS lobectomies in Iceland over the 4-year study period. Surgeon 2, who had several years of experience with VATS lobectomy, started working in Iceland 2020. VATS: video-assisted thoracoscopic surgery.

The average operative time for each year and for surgeons 1 and 2 who performed all of the operations are shown in Fig. 2. The mean operative time was 142 min (range: 47–280 min) decreasing significantly over the 4-year period, reaching 124 min in 2022 (*P* = 0.031). Surgeon 1, performing his first VATS lobectomy in 2019, had an average operative time of 152 min, decreasing from 168 to 132 min during the study period (*P* = 0.029). Surgeon B, who had been performing VATS lobectomies for 7 years in Sweden, had an average operative time of 107 min, and his operating time did not change during the study period.

**Figure 3:**
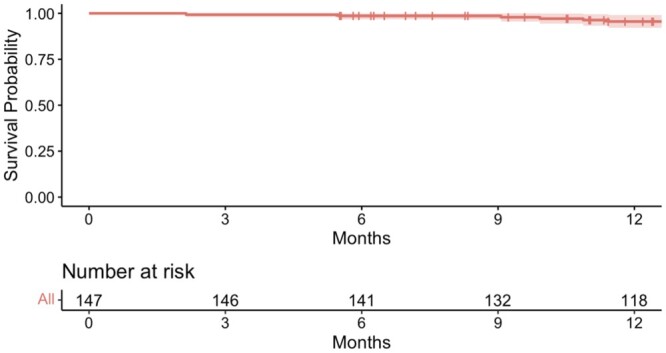
The overall survival for all 147 VATS lobectomy patients in Iceland, 2019–2022. VATS: video-assisted thoracoscopic surgery.

Overall survival is shown in Fig. 3, with a Kaplan–Meier graph. All patients survived the operation with a 0% 30-day and hospital mortality. Ninety-day mortality was 0.68% and 1-year survival was 95.6%.

## DISCUSSION

In this nationwide retrospective cohort study on 147 consecutive NSCLC patients treated for curative resection, we investigated the implementation of VATS lobectomy in our low-volume single centre. Although the introduction of this technically challenging procedure happened overnight, we experienced low complication rates, all patients survived 30 days, short postoperative stay and low 1-year mortality. Furthermore, the mean operative time decreased significantly during the period, reaching around 2 h in 2022. We believe that our findings support that VATS lobectomy can be implemented safely in a low-volume centre without compromising patient safety and quality of care.

All patients survived 30 days postoperatively, in line with prior studies on VATS lobectomy [15], but similarly our own long experience with anterolateral thoracotomy, where mortality has been below 1% [2]. Due to insufficient follow-up time, we are unable to draw conclusions on longer-term outcomes; however, our 95.6% 1-year mortality is comparable to previous reports from high-volume centres [6, 16, 17].

Our overall rate of complications was relatively low, usually ranging between 6% and 34% in prior studies. However, this fluctuation may be dependent on varying definitions for complications [18–20]. Importantly, most of the complications observed were minor and only 2.7% of the patients (*n* = 4) sustained a major complication, most often myocardial infarction (1.4%) and reoperation for bleeding (1.4%). For comparison, other studies have reported major complication rates of around 8% [18, 20, 21]. Persistent air leakage extending 7 days was the most common minor complication (8.8% and 12.1% after 5 days) and is the most common cause of prolonged hospital stay after lung cancer surgery [22]. The median postoperative stay in the current study was 4 days, which is in line with many other studies [6, 17, 23], although shorter times (2–3 days) have been reported [24, 25]. Notably, 31.3% and 12.2% of our patients were discharged on postop days 2 and 3, respectively. For comparison, an Icelandic study on 493 lobectomies performed with anterolateral thoracotomy between 1991 and 2014 reported 17.2% air leakage over 7 days and the median postoperative length of stay was 9 days [2]. Although the time periods of these 2 studies differ, the median postoperative stay has shortened substantially since VATS lobectomy was introduced, presumably driven by the much lower rates of air leakage associated with VATS.

Our findings are comparable to an Italian study that reported short-term outcomes of VATS lobectomy, all performed by a single surgeon with previous training and work experience in a high-volume centre. Although the operative time was longer in the small thoracic unit in the study, the median postoperative stay was 4.5 days, compared to 4.1 days in the high-volume centre, but short-term outcomes were otherwise very similar, including intraoperative and 30-day mortality [23].

The mean operative time for the whole study period was 142 min, all of the operations being performed by 2 senior surgeons, regularly performing pulmonary and cardiac procedures. Surgeon 1 had no prior experience in VATS lobectomy when the study started, except for 2 months of VATS lobectomy training in high-volume centres in Sweden and Denmark, and performed all of the 33 cases during the first year of the study. Surgeon 2 started working part time in Iceland in early 2020 and performed 33 of the 147 cases (22%), but he had 7 years of prior experience with VATS lobectomies in a high-volume centre in Sweden. The median operative time for surgeon 1 decreased significantly after the first year, or from 168 min to 152 and 132 min in the last 2 years, respectively (*P* = 0.029). The learning curve for surgeon 1 therefore seems to be in line with the 35–50 operations frequently reported in the literature for VATS lobectomy, although these numbers usually originate from high-volume centres [8, 10, 11].

Today, VATS training is widely incorporated in thoracic surgery training, and therefore, a growing number of centres in the future will have surgeons equipped with VATS lobectomy experience [26]. Importantly, starting a VATS lobectomy program does not only require experienced surgeons, but also equipment, such as a thoracoscopy module with high-definition imaging and special endoscopic surgical instruments. This investment in tools, especially endoscopic staplers, is costly and one of the main reasons that the implementation of VATS has been delayed, especially in low-volume centres. Furthermore, training of other personnel, such as operating nurses and anaesthetists is also of vital importance, as is training of nurses on the wards, if ERAS fast-track principles are to be followed [27]. Although the initial cost of VATS is higher when compared to thoracotomy, VATS lobectomy has been shown to be cost-effective in numerous studies, as postoperative hospital stay is shortened, and readmissions are fewer [28–30].

### Limitations

One of the main limitations of this study is the retrospective design, as well as potential lack of documentation on the patients’ history and postoperative care. The strength of the study is that the cohort consists of patients from a whole population, all of whom were operated on in a single centre by 2 surgeons, reducing the risk of institutional bias. Importantly, during the study period, all NSCLC cases were discussed on a Tumour Board, before and after surgery, and all patients were operated on in Iceland and no patients were operated on overseas.

## CONCLUSIONS

This nationwide cohort study supports that the implementation of VATS lobectomy in small low-volume centres without prior experience in advanced anatomical VATS resections is possible within a short time frame, importantly not impacting on patient safety and with favourable short-term outcomes that are comparable to larger high-volume centres. We hope our experience will pave the way for more low-volume centres to introduce VATS lobectomy.

## Data Availability

The data underlying this article will be shared on reasonable request to the corresponding author. **Viktor Asbjornsson:** Conceptualization; Data curation; Formal analysis; Investigation; Methodology; Writing—original draft; Writing—review & editing. **Gyda Johannsdottir:** Data curation; Writing—review & editing. **Daniel Myer:** Data curation; Formal analysis; Writing—review & editing. **Thorri Geir Runarsson:** Formal analysis; Writing—review & editing. **Leon Arnar Heitmann:** Formal analysis; Writing—review & editing. **Gudrun N. Oskarsdottir:** Writing—review & editing. **Per Martin Silverborn:** Writing—review & editing. **Henrik Jessen Hansen:** Writing—review & editing. **Tomas Gudbjartsson:** Conceptualization; Project administration; Resources; Supervision; Visualization; Writing—review & editing. Interdisciplinary CardioVascular and Thoracic Surgery thanks Georges Decker and the other anonymous reviewers for their contribution to the peer review process of this article.

## ABBREVIATIONS

NSCLC: Non-small-cell lung cancer
SD: Standard deviation
VATS: Video-assisted thoracoscopic surgery
